# Schirrous Affection of the Brain

**Published:** 1826-05

**Authors:** R. Wade

**Affiliations:** Member of the Royal College of Surgeons, and Apothecary to the Westminster General Dispensary.


Art. IV.—*Schirrous Affection of the Brain.* By R. Wade, Member of the Royal College of Surgeons, and Apothecary to the Westminster General Dispensary.

William Painter, fifty-eight years of age, of a robust form and florid complexion, was attacked, towards the end of October last, with paralysis of the right side; for which he was bled largely, but without any immediate relief. On my first visit, three days after the attack, I found the patient in a comatose state, with stertorous breathing, and a slow full pulse; the pupils were much contracted, and immovable. When spoken to very loudly, and requested to put out his tongue, he would make an attempt to do so, but had not the power. When asked if he felt pain, he placed his hand on the left side of his head. Active depletion was adopted. In a fortnight he recovered his speech, and in a month was so far convalescent, as to be able to walk down stairs with but little assistance.

Notwithstanding strict injunctions to the contrary, he returned to his former habits, drinking his usual allowance of porter, and eating heartily of animal food: a relapse was the consequence, and in a few days he had a second attack of paralysis. His vision then became very imperfect. Depletion was again had recourse to, but not with the same success as before. He soon recovered his speech, but obtained only a partial use of the muscles of the affected side. When left to himself, he was either in a lethargic state, or talking in an incoherent manner. He would answer a question when spoken to loudly; and at times his conversation was rational, except that he had always a disposition to wander from one subject to another. Since the last attack, he has never been able to distinguish objects with any accuracy: a hat, for example, would be mistaken for a hand; and, in fact, a candle was the only object which, when placed before him, he could rightly name, and that not always.

When other means had failed in affording relief, mercury was tried, and appeared to produce a decided improvement during the first six weeks: his vision improved, he became less comatose; indeed, all the symptoms were ameliorated. Although given in large doses, the mercury scarcely produced any effect on his mouth. This remedy, after a time, appeared to lose its beneficial influence, and was consequently discontinued. The coma increased; the sphincters of the bladder and rectum at length lost their power. He continued getting gradually worse, and died on the 9th of March.

No convulsions occurred during his illness, and the pupils were invariably contracted. For the last two months, he scarcely complained of any pain in the head. The pulse, until the last fortnight, was quite regular, being seldom above, and generally under seventy. The tongue was quite clean, and no irritability of stomach observed, although the bowels were very torpid.

I have since learned from the patient's friends, that, in the year 1810, he was employed as clerk in the Post-office at Portsmouth, and was then considered to be of sound intellect, with an excellent memory. In consequence of intemperance, he was soon obliged to leave this situation, and became agent to a newspaper office. Having occasion to go to London in 1813, he fell from the coach, and was obliged to return to Portsmouth. His conduct was then observed to be extremely inconsistent; but this, by his family, was attributed more to drinking than to the effects of the fall. Amongst other inconsistencies, he would order several joints of meat to be sent home at one time, with a corresponding quantity of vegetables. His memory was very imperfect, but no pain in the head was complained of. A few months after his fall, Mr. Painter, in consequence of misfortunes, was obliged to go to the Fleet, where he was detained ten months, during which time he was in a very desponding state. Soon after his return from confinement, he married a second time, and his wife has informed me that, ever since his marriage, he had at times complained of giddiness in the head, but seldom of much pain; that he would generally, if left unemployed, feel a strong disposition to sleep, which had much increased of late years; he would sometimes fall asleep immediately after breakfast. His memory was so bad, that, if asked to go to a shop for two articles, he would be sure to forget one of them. He was very seldom able to hold a connected conversation for any length of time, and would frequently talk to himself. Independent of the occasional giddiness and defective memory, his general health had been extremely good, and he would often observe that he had never required any medicine. For some months before the paralytic affection occurred, the patient had been subject to startings in his sleep, and would sometimes awake suddenly, and begin to pray in the most earnest manner, but always in a desponding tone; then, without any obvious cause, his devotions would be succeeded by a fit of swearing. He could not be prevented from frequently rising in the middle of the night, to smoke his pipe. These are the principal facts worthy of remark.

*Sectio cadaveris.*—On removing the calvarium, which was very easily separated from the dura mater, an exostosis was observed on its internal surface, situated on the upper and middle portion of the right side of the os frontis, measuring two inches in circumference, and a quarter of an inch in thickness. All that portion of the dura mater pressed upon by the tumor was extremely thin and transparent, and had an opening in its centre, half an inch in diameter; a corresponding depression, of course, appeared in the brain. Not the slightest appearance of inflammation was observed around this part, nor did the pia mater any where exhibit the least marks of inflammation or congestion. The lateral ventricles contained about an ounce of serum. In the upper part of the left lobus cerebri, half an inch beneath its surface, a hard mass was discovered, of a light brown colour: this was readily separated, with the handle of a scalpel, from the surrounding portion of brain, which was, to some extent, of the consistence of thick cream. The tumor, when divided, resembled exactly in appearance the medullary sarcoma, as described by Mr. Abernethy; although, on examination, it was found to have nothing of the pulpy consistence so commonly observed in that disease. In its structure, it was much more like schirrus of the mammary gland; the largest portion of the tumor consisting of striæ, of a yellowish white colour, approaching in hardness to cartilage, whilst the remainder had a dark grey appearance, and was of a texture much less firm. After macerating the tumor for a few days, and comparing a section of the hardened striæ with a schirrous cardia in my possession, the structure of the one could scarcely be distinguished from the other. The tumor was about the size of a small hen's egg. With the exception of the schirrous mass, the brain was softer and less vascular in appearance than usual.

Whilst innumerable cases have been recorded, bearing ample testimony that scarcely any part of the human body is exempt from cancerous action, it would appear that the brain had been considered, by almost every author who had written on Carcinoma, as a privileged organ, happily possessing a protection against this destructive disease. In the present pathological age, carcinomatous affections of the brain are seldom heard of, yet an occasional dissection has afforded undoubted proof that this organ is susceptible of that alteration of structure termed schirrus. Although this disease affecting the brain may, in the present state of our knowledge, be regarded, comparatively speaking, as of rare occurrence, yet it is not improbable that many of the hard masses occasionally found in either of its hemispheres, and described as scrofulous, might have been found, if more minutely examined, to have been of a schirrous nature. Dr. Baillie, in his excellent work on Morbid Anatomy, does not allude to this disease. In the second volume of the Medico-Chirurgical Transactions, Mr. Copland Hutchison, describes a case in which he found, on dissection, a schirrous tumor in the left lobe of the cerebellum: he states that the man, in whom the disease existed, had been ill for many months; that he remained sensible to the last, and always gave a coherent answer to every question; but, when not spoken to, he lay nearly in a state of coma, or low muttering delirium; which symptoms, however, only occurred a few days previous to dissolution. He never had a fibre paralysed.

In the fourth volume of the Medico-Chirurgical Transactions, another case is mentioned by the same gentleman, of a somewhat similar affection. Mr. Hutchison describes this tumor as a condensed, or rather indurated, portion of the left hemisphere of the brain, of the size of a hen's egg. It was occasioned by a wound from a cutlass, six years before the patient's death, over the left parietal bone. The symptoms described are—frequent fits of stupor, at irregular intervals; during the intermissions he was perfectly sensible; his pulse, pupils, and countenance natural; appetite good; no paralysis occurred; his bowels were torpid.

The case of Mr. Painter, when compared with others which have been described, will be found to bear, in many of its symptoms, a striking resemblance to them. The digestive functions remained but little disturbed nearly to the last; the patient preserved his usual healthy appearance till within a very short time of his death; his appetite was invariably good, he was constantly asking for food.

Delpech, in describing the symptoms of this disease, has alluded to the inclination for food in very strong terms: he observes, that "the patient appears to live only to eat,"—and sleep, he might with truth have added, as drowsiness has accompanied every case yet recorded. The paroxysms of pain in the head are mentioned by him as having been very severe: in the present case, however, they were very slight; indeed, so much so, as seldom to excite the attention either of the patient or his friends; lethargy and loss of memory being almost the only symptoms observed, until within the last year, when occasional paroxysms of delirium occurred, but apparently unattended by pain.

There surely can be but little doubt, from the preceding history, that the fall from the coach in 1813 was the origin of the mischief, as the patient's intellects had never been perfect after that occurrence. It is probable that, in consequence of the injury which the brain sustained from the fall, aggravated by the patient's intemperate habits, a low degree of inflammation was established, coagulable lymph became effused, and thus formed the nidus of the schirrous action which ensued.

In conclusion, I may observe, that it is a common remark that descriptions of very unusual diseases are more curious than useful, especially when, from their very nature, they are incurable. It should be recollected, however, that the apparent rarity of the disease may arise much from our own want of observation. This may fairly be concluded, when we consider the number of causes which, unfortunately for the advancement of our profession, so frequently happen to prevent post-mortem examinations. Whenever a tolerably accurate history of a disease, however unusual, can be obtained, it surely cannot be unreasonable to suppose that some practical benefit may ultimately accrue from ascertaining its organic changes, a knowledge of which may possibly assist the diagnosis of others.

*April* 1826.

